# Stability of creatine monohydrate and guanidinoacetic acid during manufacture (retorting and extrusion) and storage of dog foods

**DOI:** 10.1111/jpn.13103

**Published:** 2019-05-09

**Authors:** Antonius F. B. van der Poel, Ulrike Braun, Wouter H. Hendriks, Guido Bosch

**Affiliations:** ^1^ Animal Nutrition group, Department of Animal Sciences Wageningen University & Research Wageningen The Netherlands; ^2^ AlzChem Trostberg GmbH Trostberg Germany; ^3^ Department of Farm Animal Health Utrecht University Utrecht The Netherlands

**Keywords:** additive, creatinine, guanidinoacetic acid, heat sterilisation, stability, uniformity

## Abstract

The stability of creatine monohydrate (CrMH), crystallised guanidinoacetic acid (GAA‐C) and granulated GAA (GAA‐G) in a moist retorted and a dry extruded dog food formulation during production and storage was investigated. Commercial food mixtures were supplemented with CrMH, GAA‐C or GAA‐G. Uniformity after mixing and retorting or extrusion was determined based on replicate samples (moist *n* = 8, dry *n* = 10). Storage stability was evaluated at 25°C/60% relative humidity for 15 months and 40°C/75% for 6 months. Foods with CrMH were analysed for creatine (Cr) and creatinine (Crn), whereas GAA‐C and GAA‐G foods were analysed for GAA concentrations. Coefficients of variation (CV) for uniformity of the additives after mixing of moist and dry pet food formulations were below 15%, and the CV was lower in processed mixtures. Recoveries after retorting and extrusion were higher for GAA‐G (79 and 99%) and GAA‐C (89 and 86%) compared to CrMH (36 and 85%) foods. In moist CrMH food, Cr concentrations re‐increased by 54% whilst Crn concentrations decreased by 39% after storage at 25°C for 15 months. With total molar Cr + Crn remaining stable throughout storage, Crn and Cr appeared to effectively interconvert. Storage of the extruded CrMH food at 25°C for 15 months resulted in a 63% decrease in Cr and a 39% increase in Crn concentration. The decrease in Cr concentration was larger at 6 months storage at 40°C compared to 15 months storage at 25°C. Both GAA‐C and GAA‐G moist and dry foods were stable during storage (<10% decrease). This study showed that GAA is highly stable during production and storage of moist and dry canine foods whilst CrMH is relatively unstable, particularly during storage. The latter makes it difficult to establish a guaranteed Cr content in finished moist retorted and dry extruded foods with CrMH.

## 1 INTRODUCTION

Creatine (Cr) is a naturally occurring constituent and essential nitrogenous compound in vertebrate animals. Cr is stored primarily in the skeletal muscle, where it is phosphorylated by ATP to phosphocreatine (PCr), which serves as a reserve store for high energy phosphate to restore ATP from ADP (Beitz, 2004; Hageböck, Stahl, & Bader, 2014; Wyss & Kaddurah‐Daouk, 2000). As such, PCr maintains ATP levels and prevents AMP formation and further breakdown to reactive oxygen species (Sahlin, Cizinsky, Warholm, & Högberg, 1992). Synthesis of Cr in the body occurs from the amino acids, glycine and arginine, yielding guanidinoacetic acid (GAA), which is further methylated to Cr by S‐adenosyl‐methionine. In human sports nutrition, Cr is well accepted to improve physical performance. Although the NRC (2006) concluded that there is currently no evidence for any benefit of Cr addition to the diet of exercising dogs, a recent study reported benefits from adding a source of Cr to dog food. Including GAA, the endogenous precursor of creatine as an additive in a moist food of active Foxhound‐Boxer‐Ingelheim Labradors (0.3 mmol/kg body weight) for 30 days increased Cr concentrations in blood serum and muscle tissue and reduced body fat content (Dobenecker & Braun, 2011). The authors concluded that dogs efficiently utilise and metabolize GAA to Cr. In addition, an elevated Cr intake has also been reported to accelerate skeletal muscle recovery after prolonged immobilization, slow down muscle wasting in certain myopathies and improve cognitive performance (Hespel et al., 2001; Louis et al., 2003; McMorris et al., 2007; Tarnopolsky et al., 2004). The value of Cr in the diet of geriatric dogs and possibly cats suffering from cognitive dysfunction, cachexia and sarcopenia is largely unknown.

Animal‐derived food or feed ingredients such as muscle tissue, organs and rendered meals are natural sources of Cr. As such, pet foods based on these ingredients may also contain Cr. When investigating the Cr concentrations in meat, offal or commercial dog food, Harris, Lowe, Warnes, and Orme (1997) reported levels between 27.8 and 32.3 mmol/kg (3.6–4.3 g/kg) in uncooked chicken, beef and rabbit meat, which are well in the range for values reported for muscle. However, the authors found no PCr and low amounts of Cr in eight moist retorted (0.5–2 mmol/kg) and four dry extruded (0.5–4 mmol/kg) commercial dog foods. Creatinine (Crn) concentrations were generally higher than Cr (10 of the 12 foods), and the authors concluded that in the canned dog foods, as well as in dried meat samples and rendered meat meal, Cr is degraded to a variable extent to Crn. When investigating the Cr supply by different types of dog food, as compared to prey, Dobenecker and Braun (2015) confirmed the findings by Harris et al. (1997) as they found mean values of 9.6 and 10.3 mmol/kg dry matter (DM) for Cr and Crn, respectively, in commercial moist dog foods (*n* = 32) and 0.6 and 3.5 mmol/kg DM for Cr and Crn for commercial dry dog foods (reported values in mg/kg DM transferred to mmol/kg DM). Overall, Dobenecker and Braun (2015) showed that the highest Cr supply per MJ of diet is provided by unprocessed food, especially prey and that processed dog foods and ingredients (meat and bone meal, dry and moist foods) contained significantly less Cr and more Crn. As already assumed by Harris et al. (1997), the high Crn levels were attributed to degradation of Cr due to heat treatment, whilst the increased use of vegetal matter in the formulation of especially dry commercial pet foods, likely adds further to their extremely low Cr concentration.

The synthetic product Cr monohydrate (CrMH), the primary dietary additive form of Cr (Jäger, Purpura, Shhao, Inoue, & Kreider, 2011), has been intensively studied and described as a dietary additive for humans and livestock animals (Harris & Lowe, 1995; Lowe, Murphy, & Nash, 1998; Persky & Brazeau, 2001; Zhang et al., 2014). Due to the instability of CrMH in aqueous and acidic solutions as well as at high temperatures yielding the physiologically inactive degradation product Crn (Cannan & Shore, 1928; Hahn & Bakan, 1920; Jäger et al., 2011), its use as a direct animal feed additive is limited (Baker, 2009). There have been alternative forms of Cr (e.g., ethyl ester, malate, pyruvate, gluconate) marketed as additives in the late 1990s that were not shown to be more effective or safer compared to CrMH (Jäger et al., 2011). Alternatively, Cr can be supplied from its endogenous precursor GAA added to dog foods as indicated above. Stability of supplemented Cr or GAA in pet foods is important as its manufacture (extrusion, retorting) involves thermal and shear processing and potentially extensive periods of storage.

The present study was conducted to determine the stability of two preparations of GAA (granulated and crystallised) and CrMH in a moist and a dry dog food formulation during production and storage. Hereto, stability after retorting and extrusion, and after subsequent storage at 25°C up to 15 months and 40°C up to 6 months were studied. In addition, we evaluated mixing uniformity for each of these supplements in the food mixtures.

## 2 MATERIALS AND METHOD

### 2.1 Supplements

Synthetic CrMH (Creapure^®^, 89.7% in wet study and 90.2% in dry study Cr) and GAA as a granulated (GAA‐G, CreAMINO^®^, 96.0% GAA for moist food study and 97.8% for dry food study) or crystalline (GAA‐C, 99.9% GAA for moist food study and 99.8% for dry food study) product were obtained from AlzChem AG.

### 2.2 Moist retorted food

#### 2.2.1 Food manufacture

One day prior to the experiment, 40 kg of a commercial, moist canine food mixture intended for retorting (heat sterilisation) was purchased from Partner in Pet Food and transported on ice, to Wageningen University & Research (Wageningen, The Netherlands) and stored at −20°C. The formulation was not disclosed but contained (in decreasing level of inclusion) the following: meat and animal by‐products, cereals, minerals, vegetable by‐products and various sugars. The analysed chemical composition of the mixture is provided in Table 1. Since the mixture contained meat and animal by‐products, the base mixture provided baseline values for Cr and Crn levels. The moist canine mixture was processed as such (base mixture) or mixed (after defrosting and acclimatisation for 4 hr) in a temperature‐controlled environment (~15°C) with one of the three additives (treatment mixtures). The inclusion level for all test materials was 1 g/kg food as‐is. The mixtures of test diets were obtained by adding 3.2 g test material in four portions to 3.2 kg of base diet with a spoon prior to mixing in the Bestron planetary mixer (4.6 L, type DHA3470). The base mixture and each of the three treatment mixtures were thoroughly mixed in the mixer for 120 s at average speed setting before being used to manually fill (400 ± 2 g) 24 individual lacquered cans (Ø 72 mm × 110 mm) per treatment. After cleaning the top, cans were sealed using a rotary seamer (Lanico‐Maschinenbau, Type V10 ARFE, Otto Niemsch GmbH). To prevent cross‐contamination, the apparatus was thoroughly cleaned between the preparations of the mixtures.

**Table 1:** Chemical composition (g/kg dry matter) a of the unprocessed moist and dry canine food mixtures

Component	Moist food mixture	Dry food mixture
Dry matter (g/kg as‐is)	181	917
Crude protein	425	316
Crude fat	266	70
Crude fibre	16	
NDF		86
Ash	138	90

Unless otherwise stated.

#### 2.2.2 Mixing uniformity and matrix stability

Mixing uniformity was based by randomly selecting eight cans per treatment and analysing each for the Cr and Crn for the canned base and CrMH mixtures or GAA for the canned GAA‐G and GAA‐C mixtures. Dry matter content and pH were also measured in these selected cans. The mixing uniformity was performed for both unprocessed and retorted foods (see below) in order to calculate recovery after retorting and assess retorting stability.

#### 2.2.3 Retorting stability

Retorting stability of the three test compounds CrMH, GAA‐G and GAA‐C was evaluated by relating mean values of the uniformity samples (*n* = 8) to mean values of the samples after retorting (*n* = 8). Heat sterilisation of 16 cans per treatment was achieved in a batch steam retorting system (autoclave) with the cans of all treatments autoclaved in one batch at the facilities of Wageningen University & Research. Eight cans were randomly assigned as retorting stability samples and storage stability samples respectively. The measured process conditions for temperature/time settings were heating up to 126°C (36 min) followed by iso‐thermic heating at 126°C during 60 min and cooling down for 6.5 min. The process temperature was continuously recorded using a thermal‐/pressure‐sensitive data logger (PicoVACQ PT wireless data logger, TMI‐ORION) having a PT1000 temperature sensor located inside one of the cans. Following heat sterilising, cooling of the cans took place inside the autoclave with water and, after opening the autoclave and removing the cans, under ambient temperature. Cans were sent on ice to AlzChem AG for either immediate analysis for retorting stability assessment or placed in climatic chambers for storage stability evaluation. All unprocessed and retorted cans were stored and transported at −20°C pending analyses.

#### 2.2.4 Storage stability

The retorted cans from the base matrix or containing CrMH, GAA‐G and GAA‐C were placed in climatic chambers (KFB 720 type, Binder GmbH) at AlzChem AG. One can per time point (*n* = 1) was taken out of the chamber and analysed according to the procedures described below. Time points were 3, 6, 9, 12 and 15 months for storage at 25°C/60% humidity and 1, 3 and 6 months for storage at 40°C/75% humidity. Storage temperature was controlled within ± 2°C of its target value. At the designated storage time, one can per treatment was used to determine Cr, Crn (base and CrMH mixtures) and GAA (GAA‐G and GAA‐C mixtures) concentrations, DM content and the pH. For time point 0, the mean values for the retorting stability were used. Samples were shipped and analysed immediately after the experiment and kept at all times at −20°C pending analyses.

### 2.3 Dry extruded food

#### 2.3.1 Food manufacture

One, ~1,300 kg batch of a commercial, ground, dry mixture formulated for dogs was purchased (Vobra BV). The dry mixture contained the following (in decreasing level of inclusion): poultry meal, maize, wheat, rice, rice bran, beet pulp, vitamin/mineral pre‐mix, soy lecithine, NaCl, antioxidant/salmonella agent and CaPO_4_. The analysed chemical composition of this mixture is provided in Table 1. Four mixtures were prepared (300 kg each): a base mixture (no additive) and three experimental mixtures with either CrMH, GAA‐G or GAA‐C added. Inclusion rates were 2.8 g/kg as‐is for CrMH (2.53 g/kg Cr) and 2.2 g/kg as‐is for both GAA mixtures. To facilitate mixing, the additive was first mixed (Forberg F60 paddle‐shift mixer) for 3 min with ~30 kg of the base mixture. The final mixture was then prepared by mixing the 30 kg with the remaining 270 kg for 15 min in a vertical, conical screw‐type mixer, type Vrieco (Hosokawa Micron BV). Between batches, all processing equipment was thoroughly cleaned to avoid contamination between batches. All mixing steps were performed at ambient temperature.

#### 2.3.2 Mixing uniformity and matrix stability

The experimental mixtures were used to fill 12 paper bags of 25 kg. From each mixture, the first and the final bag were disregarded for reason of process uncertainty and the remaining bags of each mixture were sampled (250 g) using a clean sampling spear for determination of mixing uniformity (*n* = 10). For the base ingredient mixture without the test supplements, only one sample was taken and analysed as the mixture was deemed homogenous and uniformity was expected to be high.

#### 2.3.3 Extrusion stability

Extrusion stability of base food as well as CrMH, GAA‐G and GAA‐C in experimental foods was evaluated by relating mean values derived from mixing uniformity evaluations with mean values derived from the samples after extrusion (*n* = 10). Before mixtures were extruded, the single screw extruder (AL150, Almex) was warmed‐up by processing a surplus (~100 kg) of the commercial, ground, dry mixture for 15 min. The remaining content of the 10 bags for each mixture (base, CrMH, GAA‐G and GAA‐C) was manually transferred into a 600 l storage bin located above the conditioner of the extruder at the feed processing facilities of Wageningen University & Research. Via a feeder screw, the mixtures were fed into the conditioner. The mixtures were conditioned with water (80 g/kg) and saturated steam (30 g/kg), and following the release from the conditioner they were extruded. The residence time in the conditioner was estimated to be 20–25 s. The extruder had a screw configuration of L/D = 8 with material residing in the extruder for ~10 s, and a die size of 6 mm with four orifices was used. The extruder capacity was ~400 kg/h, and product temperature at the end of the die was between 125 and 130°C as determined with a Pt100 temperature sensor. Nineteen kibble samples (10 for extrusion stability, nine for storage stability) of 250 g were collected for each extruded mixture at the extruder die at regular intervals of approximately 2 min once steady state conditions were achieved. Thereafter, kibbles were dried in a Heratherm OMH 750‐3P, forced air drier (Fisher Scientific) at 50°C for 14 hr before being packed in double wall paper bags (20 × 10 × 46.5 cm).

#### 2.3.4 Storage stability

The bags intended for extrusion storage stability were stored in temperature‐controlled climate chambers at AlzChem AG. Specifications of the chambers and storage conditions and durations were similar to the canned foods. Sampling time points at which one bag was taken out of the chamber (*n* = 1) and analysed according to procedures below were as follows: 3, 6, 9, 12 and 15 months for storage at 25°C/60% relative humidity and 1, 3 and 6 months for storage at 40°C/75% relative humidity.

### 2.4 Chemical analyses

The base mixtures for the moist and dry canine foods were analysed for crude protein (N × 6.25), crude fat, crude ash, crude fibre (moist food mixture), neutral detergent fibre (dry food mixture) and DM using standard methods as described previously (van Rooijen et al., 2014). For pH determination, 10 g of the sample was weighed into a 100‐ml volumetric flask and made up to volume with distilled water. The resulting suspension was stirred for 5 min. The pH was determined directly in the suspension using a calibrated pH meter (691 pH Meter, Metrohm). For the analyses of Cr, Crn and GAA, extracts from the unprocessed mixtures and retorted or extruded foods were prepared. Samples were homogenised thoroughly in a laboratory mill, and 10 g of each sample was accurately weighed into a 500‐ml volumetric flask. Water (350–400 ml) was added and the suspension sonicated four times for 5 min each with hand mixing in between. Flasks were made up to volume before an aliquot of the suspension was filtrated through a 0.45‐µm membrane filter and a SPE cartridge and analysed immediately for GAA, Cr and Crn. An ion chromatography system (Dionex ICS3000 or 5000) with gradient pump and variable wavelength detector were used for GAA and Cr analyses, the Hypersil Hypercarb column (4.6 × 100 mm) was obtained from Thermo (P/N 35007‐104630), and the Aminopac PA1 column (4 × 250 mm) and pre‐column Aminopac PA1 (4 × 50 mm) were obtained from Dionex (P/N 37022 and P/N 37023). For Crn analyses, the two Hypercarb columns (7 µm, 4.6 × 100 mm, in a serial arrangement) were obtained from Thermo (P/N 35007‐104630). Peaks were quantified using Cr, Crn and GAA standards purchased from Sigma‐Aldrich. The DM content was also determined in these samples. Samples were thoroughly homogenised in a laboratory mill after which 1 g subsample was accurately weighed into a weighing boat and dried in a drying oven (VT 6060‐BL, Thermo Fisher Scientific) to a constant weight at 105°C for 3 hr. After cooling in a desiccator to ambient temperature, the sample was weighed again and loss on drying was determined by calculation.

### 2.5 Data analyses

Mixing uniformity was based on mean and standard deviation (*SD*) of analysed replicate samples and expressed as coefficient of variation (CV; *SD*/mean × 100%) for each unprocessed mixture and for each retorted and extruded food. The mean values were also used to calculate retorting and extrusion stability, which was expressed as mean value after processing/mean value before processing × 100%. Storage stability was evaluated by calculating the change in the concentration of the test materials over time. Linear regression analysis was used to derive the relationship between Cr + Crn (in mmol/kg DM) over time of storage.

## 3 RESULTS

### 3.1 Pre‐processing

The average Cr and Crn concentrations of the moist, base mixture before heat sterilisation, were, respectively, 1.65 and 0.05 g/kg DM, and the concentrations in the mixtures supplemented with CrMH were on average 6.26 g/kg DM for Cr and 0.08 g/kg DM for Crn (Table 2). The commercial dry base mixture contained on average 0.12 g/kg DM Cr and 0.92 g/kg DM Crn and the mixture to which CrMH was supplemented contained on average 2.85 g/kg DM Cr. Wet and dry food mixtures supplemented with GAA‐G or GAA‐C contained on average similar concentrations of GAA. All pH values measured of the moist canine food mixtures before retorting ranged between 5.7 and 6.6 (data not shown).

**Table 2:** Uniformity (%) and recovery (%) of creatine, creatinine and guanidinoacetic acid (GAA) concentrations (g/kg dry matter) after mixing creatine monohydrate (CrMH) or GAA in granulated (GAA‐G), or crystallized (GAA‐C) form in unprocessed moist and dry canine food mixtures and after heat treatment of the moist (retorting) and dry (extrusion) food mixtures. (*n* = 8 for moist and *n* = 10 for dry foods)

Food	Mixture	Uniformity after						Recovery^a^
		Mixing			Heat treatment^a^			
		Mean	*SD*	CV	Mean	*SD*	CV	
Moist	Base							
	Creatine	1.65	0.26	15.6	0.54	0.01	2.5	33
	Creatinine	0.05	0.01	13.6	0.73	0.02	2.5	1612
	Base + CrMH							
	Creatine	6.26	0.38	6.1	2.26	0.12	5.2	36
	Creatinine	0.08	0.01	6.3	2.95	0.13	4.4	3,656
	Base + GAA‐G							
	GAA	5.64	0.57	10.0	4.65	0.25	5.5	79
	Base + GAA‐C							
	GAA	5.91	0.88	14.8	4.99	0.22	4.5	89
Dry	Base							
	Creatine	0.12	–	–	0.12	0.00	2.0	99
	Creatinine	0.92	–	–	0.78	0.01	0.7	85
	Base + CrMH							
	Creatine	2.85	0.06	2.2	2.41	0.02	0.8	85
	Creatinine	0.66	0.01	1.5	1.12	0.01	0.8	170
	Base + GAA‐G							
	GAA	2.35	0.08	3.5	2.31	0.05	2.1	99
	Base + GAA‐C							
	GAA	2.37	0.02	0.9	2.03	0.37	18.2	86

Abbreviations: *SD*: standard deviation; CV: coefficient of variation.

a Recovery after heat treatment as a percentage of the value in the uniformity study.

### 3.2 Uniformity after mixing and heat treatment

At the time of mixing of the CrMH, GAA‐G and GAA‐C into the unprocessed base mixture for the moist canned food, particles of variable sizes were observed, ranging from small to relatively large (4–6 cm) particles. Relatively high CV values for all moist food mixtures were observed with CV values ranging from 6.1% to 15.6% (Table 2). The CV values after retorting were lower compared to their unprocessed counterparts with values recorded between 2.5% and 5.5%.

Coefficients of variation were generally lower for the dry mixtures (Table 2) compared to the moist mixtures. The CV of GAA‐C in extruded foods was unexpectedly high (18.2%) and considerably higher than that of the mixture containing GAA‐G (2.1%). The same mixture was therefore extruded in a new run and obtained uniformity after extrusion was 4.3% (*n* = 10 samples) with a mean ± *SD* of 2.31 ± 0.10 g/kg DM.

### 3.3 Stability during retorting and extrusion

Upon heat sterilisation of the base moist food, the concentrations of Cr decreased to 0.54 and Crn increased to 0.73 g/kg DM (Table 2). After retorting of the moist CrMH food, the average Cr concentrations were also decreased (2.26 g/kg DM) and those of Crn increased (2.95 g/kg DM), which correspond to a recovery (relative to initial values) of 36% for Cr. Relative to the CrMH additive, the two GAA product forms showed a higher recovery (79% for GAA‐G and 89% for GAA‐C) upon heat sterilisation. The pH values of the moist canine foods were increased after retorting and ranged between 7.1 and 7.3 (data not shown).

Processing the CrMH, dry food mixture via extrusion resulted in 85% recovery of the Cr whilst Crn concentrations increased from 0.66 to 1.12 g/kg DM (Table 2). In the base mixture, there was no decrease in Cr concentrations after extrusion whilst Crn decreased from 0.92 to 0.78 g/kg DM. After extrusion, the GAA‐G and GAA‐C additives showed recoveries of 99 and 86% (Table 2). The latter value for recovery would be 97% in case the results of the second extrusion run were used (see above).

### 3.4 Stability during storage

**Figure 1:**
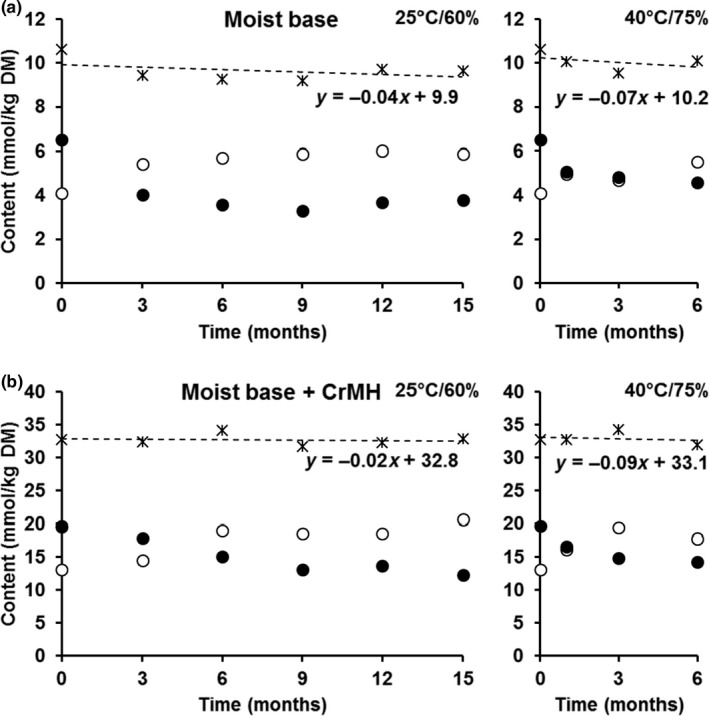
Changes in creatine (○), creatinine (⚫) and creatine + creatinine (ᚼ) concentrations (mmol/kg dry matter [DM]) during storage at 25°C with 60% humidity (left panels) and at 40°C with 75% humidity (right panels) for the moist retorted canine food without (base; panels A) or with creatine monohydrate (base + CrMH; panels B). Values for the CrMH diet are corrected for creatine and creatinine content in the base moist mixture at each time point. (*n* = 1 except for initial value, see Table 2)

Table 3 shows the concentrations of Cr, Crn and GAA in the moist retorted and dry extruded foods stored at 25°C with 60% relative humidity for up to 15 months and 40°C with 75% relative humidity for up to 6 months. For the moist base diet and the moist base diet supplemented with CrMH, concentrations of Cr increased and Crn decreased during the first 6 to 9 months of storage at 25°C and then stayed fairly constant. For storage at 40°C during 6 months, concentrations of Cr were also found to increase and those for Crn to decrease and these changes were already observed after 1 months of storage. The equilibrium between Cr and Crn during storage is illustrated in Figure 1. The concentrations are expressed on a molar basis, and the CrMH food is corrected for the background Cr and Crn from the base food to illustrate the response of the additive. For the CrMH, Crn is reconverted into Cr at a slower rate than the Crn in the base food at 25°C. For both foods, the total molar amounts of Cr and Crn seemed to remain stable with the slope of the regression lines being close to 0. Granulated GAA in the moist food was stable upon 15 months of storage at 25°C. Also at 40°C, GAA‐G in the moist food had a recovery of close to 100% after 6 months. Recrystallised GAA also showed a good stability (>90%) upon 15 months of storage at 25°C with a recovery of >95% after 6 months of storage at 40°C in the moist food. The pH in the moist foods remained stable with values between 7.0 and 7.2 for storage at 25°C and 6.7–7.3 for storage at 40°C (data not shown).

**Table 3:** Changes in creatine, creatinine and guanidinoacetic acid (GAA) concentrations (g/kg dry matter) during storage at 25°C with 60% humidity and 40°C with 75% humidity of a moist retorted and a dry extruded canine food without (base) or with creatine monohydrate (CrMH) or GAA in granulated (GAA‐G) or recrystallised (GAA‐R) form. (*n* = 1 except for initial value, see Table 2)

Food	Mixture	Initial	Storage temperature and time (months)							
			25°C					40°C		
			3	6	9	12	15	1	3	6
Moist retorted	Base									
	Creatine	0.54	0.71	0.75	0.77	0.79	0.77	0.65	0.62	0.72
	Creatinine	0.73	0.45	0.40	0.37	0.42	0.43	0.57	0.55	0.52
	Base + CrMH									
	Creatine	2.26	2.61	3.24	3.21	3.23	3.48	2.76	3.16	3.05
	Creatinine	2.95	2.48	2.11	1.86	1.97	1.81	2.45	2.23	2.13
	Base + GAA‐G									
	GAA	4.65	4.30	4.76	4.24	4.47	4.72	4.72	4.80	4.69
	Base + GAA‐C									
	GAA	4.99	4.91	5.18	4.56	4.64	4.52	4.95	5.97	4.75
Dry extruded	Base									
	Creatine	0.12	0.15	0.20	0.21	0.23	0.22	0.20	0.22	0.20
	Creatinine	0.78	0.80	0.71	0.61	0.74	0.54	0.61	0.57	0.51
	Base + CrMH									
	Creatine	2.41	1.97	1.53	1.31	1.21	1.11	1.13	0.91	0.79
	Creatinine	1.12	1.53	1.86	1.86	1.99	2.10	0.23	0.23	1.70
	Base + GAA‐G									
	GAA	2.31	2.19	2.30	2.30	2.31	2.21	2.32	2.26	2.15
	Base + GAA‐C									
	GAA	2.03	1.85	1.97	1.96	1.98	1.99	2.00	1.92	1.92

In contrast to the moist foods, the Cr concentration in the CrMH kibbles showed a decrease during storage at 25°C with values at 15 months being 46% of the initial values (2.41 g/kg DM) whilst a concomitant increase in Crn concentration was recorded. A similar trend was observed at 40°C although values were recorded to be decreased quicker and further than those for storage at 25°C. Concentrations of GAA in kibbles supplemented with GAA‐C and GAA‐G remained fairly stable during storage under both conditions, though some variation was observed at several time points.

## 4 DISCUSSION

### 4.1 Pre‐processing

The ingredients used for the formulation of the moist and dry mixtures were typically those used in pet food manufacture, and their nutrient contents were within the range expected for commercial moist and dry dog foods (Hand, Thatcher, Remillard, Roudebush, & Novotny, 2010). The Cr and Crn concentrations of the moist, base mixture before heat sterilisation were within the range reported for raw single animal tissues commonly used in dog rations (0.54–6.77 and 0.03–0.17 g/kg DM for Cr and Crn, respectively) reported by Dobenecker and Braun (2015). The average Cr concentrations in the moist mixture with CrMH (6.26 g/kg DM, Table 2) were close to the expected (i.e. formulated) value of 6.43 g/kg DM based on the inclusion rate, Cr content of the additive and the moisture level of the mixture. Similarly, the observed average Cr concentrations for the dry mixture with CrMH were close to the expected value (2.84 g/kg DM). For the observed average GAA concentrations, values were also close to the expected values in moist and dry food mixtures for both GAA‐G (5.64 and 2.30 g/kg DM respectively) and GAA‐C (5.70 and 2.36 g/kg DM respectively).

### 4.2 Uniformity after mixing and heat treatment

Mixing uniformity is an important property of a dietary additive in order to ensure that the additive is ingested in the correct amount compared to other nutrients. An acceptable CV for mixing uniformity is ~10% (AFIA, 1994; Behnke & Beyer, 2002). The heterogeneous nature of the unprocessed base mixture for the moist canned food complicated homogenous sampling and largely contributed to relatively high CV values for the moist food mixtures. The lower CV values after retorting were possibly due to a particles size reduction in the base matrix during cooking. Mixability of the three additives in the dry mixtures was high (i.e. low CV values) indicating that segregation was limited. Overall, the studied additives had generally a good (<10%) mixing uniformity in the moist retorted and dry extruded dog foods.

### 4.3 Stability during retorting and extrusion

Creatine has been reported to be unstable in high moisture products or aqueous solutions and may be degraded into Crn (Jäger et al., 2011). This is, however, dependent on the moisture level, pH and temperature. The equilibrium of the reaction between Cr and Crn is favoured towards Cr at high pH and low temperature, whereas Crn is favoured at elevated temperatures and in acidic solutions (Edgar & Shiver, 1925; Wyss & Kaddurah‐Daouk, 2000). Approximately two‐thirds (64%) of the original Cr in the CrMH moist food underwent an intramolecular cyclisation to form Crn with the removal of 1 mole of water in the retorting process. With the slightly increased but still rather neutral pH values and pH values in the range where Cr is reported to be relatively stable (i.e. pH 6.5, 7.5; Jäger et al., 2011), the increased temperature during retorting was likely the main driver of the observed conversion to Crn rather than change in pH. The extent of conversion was in line with Harris et al. (1997), showing that boiling of different meats for up to 60 min decreased the concentration of Cr by approximately two‐thirds and increased the concentration of Crn. Unlike the Cr from the CrMH additive, the GAA, whether in granulated or recrystallized form, was more stable in the moist canine food matrix upon retorting as indicated by the much higher recovery (36% vs. 79% for GAA‐G and 89% for GAA‐C). To the authors' knowledge, there are no data in the scientific literature showing the stability of CrMH during extrusion to manufacture dry pet foods or stability of GAA during the processing of feeds or foods. We found that the Cr from CrMH was, relative to retorting the moist food mixture with CrMH, more stable during the extrusion process (85% recovery of Cr), confirming the importance of moisture for the conversion from Cr to Crn. Also for the GAA‐G and GAA‐C additives, recoveries were higher with values of 99 and 97% (in case, the results of the second extrusion run were used, see above).

### 4.4 Stability during storage

For the moist food supplemented with CrMH, the decrease of Cr and increase of Crn after retorting was in part reverted again during storage with Cr concentrations increasing by 54% whilst Crn concentrations decreasing by 39% after storage for 15 months. Furthermore, it appeared that the total molar Cr + Crn remaining stable throughout storage, indicating that Crn and Cr effectively interconvert without any losses. In aqueous solutions, the equilibrium between Cr and Crn is also affected by temperature. Edgar and Shiver (1925) reported that the proportion of Crn increases with increasing temperature in an aqueous solution. The latter was also observed here at 3 and 6 months of storage of the CrMH food, and the molar proportion of Crn was higher at 40°C compared to the 25°C (0.52 and 0.43 at 25°C and 0.45 and 0.45 at 40°C respectively). Though for the retorted base and CrMH moist foods the Cr increased during storage (to 0.77 and 3.48 g/kg DM at 15 months respectively), the concentrations prior to sterilisation were substantially higher (1.65 and 6.26 g/kg DM respectively). We estimate that approximately 26% and 12% of the initial Cr + Crn was lost, potentially due to the Maillard reaction (Uzzan, Nechrebeki, & Labuza, 2007). The observed instability of CrMH in aqueous solutions and instability at high temperatures limits the application of CrMH in the production of wet canine foods.

Storage of the extruded CrMH food at 25°C for 15 months, however, resulted in a further decrease in Cr (63%) and an increase in Crn (39%) concentrations. The decrease in Cr concentration was larger at 6 months storage at 40°C with 75% relative humidity compared to 15 months storage at 25°C with 60% relative humidity, which might indicate that temperature and relative humidity are important factors for the stability of Cr in particular dry dog foods.

In contrast to the moist foods, the Cr concentration in the CrMH kibbles decreased due to storage whilst Crn concentrations increased. Similar trends were observed for both storage conditions but changes appeared to occur quicker and further at 40°C/75% relative humidity than those for storage at 25°C/60% relative humidity. Dash, Mo, and Pyne (2002) reported that CrMH dehydrates to Cr from 97 to 125°C with anhydrous Cr undergoing intramolecular cyclisation to Crn. It is likely that the CrMH added to the dry mixture was reversed to the anhydrous form during extrusion and subsequent drying process and that during storage, cyclisation occurred reducing the concentration of Cr and increasing the Crn concentration. The losses in Cr found for the kibbles with CrMH imply that CrMH should be added to levels in which the loss of the functional component during storage is taken into account. Furthermore, the instability of Cr during storage implies that it may be difficult to establish a guaranteed Cr content in both moist retorted and dry extruded foods.

Both the recrystallized and granulated GAA showed a good stability (>90%) throughout the storage periods at 25 and 40°C and both in the moist retorted and in dry extruded dog foods. As such, these forms of GAA appear to better fulfil the technical requirements necessary for the application as an animal feed additive.

## 5 CONCLUSIONS

The mixing uniformity of the CrMH and granulated and crystallised GAA was in general good in moist and dry dog food formulations. The stability of granulated and crystallised GAA was high (79 and 89% for GAA‐G and GAA‐C respectively) compared to CrMH (36%) during the retorting of moist and more similar during extrusion of dry canine foods (85, 99, and 86% respectively). Subsequent storage of the canned retorted food and extruded kibbles also indicated that granulated and crystallised GAA were more stable compared to CrMH. Part of the Cr in the moist foods that was reduced in concentration due to retorting was recovered due to interconversion from Crn to Cr during storage. For dry foods, Cr concentrations appeared to decrease during storage. Stability during storage of the three additives was generally higher in dry extruded compared to moist retorted dog foods.

## CONFLICT OF INTEREST

U. Braun is an employee of AlzChem Trostberg GmbH.
